# Interaction of AIM with insulin-like growth factor-binding protein-4

**DOI:** 10.3892/ijmm.2015.2262

**Published:** 2015-06-26

**Authors:** QIANG YOU, YAN WU, NANNAN YAO, GUANNAN SHEN, YING ZHANG, LIANGGUO XU, GUIYING LI, CYNTHIA JU

**Affiliations:** 1 Key Laboratory for Molecular Enzymology and Engineering of the Ministry of Education, College of Life Science, Jilin University, Changchun, Jilin 130012, P.R. China; 2 Skaggs School of Pharmacy and Pharmaceutical Sciences, University of Colorado Anschutz Medical Campus, Aurora, CO 80045, USA; 3 School of Life Science, Jiangxi Normal University, Nanchang, Jiangxi 330022, P.R. China

**Keywords:** apoptosis inhibitor of macrophage, insulin-like growth factor-binding protein-4, insulin-like growth factor, protein interaction, apoptosis

## Abstract

Apoptosis inhibitor of macrophages (AIM/cluster of differentiation 5 antigen-like/soluble protein α) has been shown to inhibit cellular apoptosis; however, the underlying molecular mechanism has not been elucidated. Using yeast two-hybrid screening, the present study uncovered that AIM binds to insulin-like growth factor binding protein-4 (IGFBP-4). AIM interaction with IGFBP-4, as well as IGFBP-2 and -3, but not with IGFBP-1, -5 and -6, was further confirmed by co-immunoprecipitation (co-IP) using 293 cells. The binding activity and affinity between AIM and IGFBP-4 *in vitro* were analyzed by co-IP and biolayer interferometry. Serum depletion-induced cellular apoptosis was attenuated by insulin-like growth factor-I (IGF-I), and this effect was abrogated by IGFBP-4. Of note, in the presence of AIM, the inhibitory effect of IGFBP-4 on the anti-apoptosis function of IGF-I was attenuated, possibly through binding of AIM with IGFBP-4. In conclusion, to the best of our knowledge, the present study provides the first evidence that AIM binds to IGFBP-2, -3 and -4. The data suggest that this interaction may contribute to the mechanism of AIM-mediated anti-apoptosis function.

## Introduction

Apoptosis inhibitor of macrophages (AIM), also known as Api6, soluble protein α or cluster of differentiation 5 (CD5) antigen-like, is a 54-kDa glycoprotein secreted by mature tissue macrophages, and it belongs to the scavenger receptor cysteine-rich domain superfamily (1–4). AIM was initially found to inhibit apoptosis of CD4^+^CD8^+^ T cells during their maturation in the thymus (3). The anti-apoptotic effect of AIM on other cells, such as natural killer T cells and macrophages, was subsequently reported (3,5–12). AIM, as a direct gene target of liver X receptors (LXRs), is induced during LXR activation by oxidized low-density lipoprotein or microbial infection (3,8,9,11,12). In turn, the induction of AIM promotes macrophage survival and accentuates the roles of these cells in the development of atherosclerosis, immunity against microbial infection and inflammatory processes (6,8–10,12). Overexpressing AIM in mice increases the survival rate and phagocytic activity of macrophages in fulminant hepatitis (6). AIM has a role in atherosclerogenesis by enhancing macrophage survival within atherosclerotic lesions (10). Results from reverse tetracycline-responsive transactivator and Api6 bitransgenic mice have shown that AIM (Api6) is a pro-inflammatory and oncogenic molecule that stimulates cell proliferation and lung tumorigenesis (13,14). Recent evidence has indicated that AIM is incorporated into adipocytes via CD36-mediated endocytosis, thereby inactivating cytoplasmic fatty acid synthase (15). Among all the biological functions associated with AIM, the anti-apoptotic function has been the most clearly demonstrated; however, the molecular mechanism by which AIM regulates apoptosis is not understood.

Insulin-like growth factor-binding proteins (IGFBPs) are an important family of secreted proteins with similar high binding affinities to IGF-I and IGF-II, and are involved in the regulation of somatic growth and cellular proliferation (16–19). At least 6 isoforms (IGFBPs 1–6) have been well characterized. The liver is the major source of circulating IGFs and IGFBPs. Hepatocytes synthesize IGFBP-1, -2 and -4, and hepatic Kupffer cells synthesize IGFBP-2 and IGFBP-3 (20,21). IGFBPs regulate IGF signaling by binding to IGF and partially masking the IGF residues responsible for type 1 IGF receptor binding (17,22,23). IGFBPs are secreted into the extracellular matrix, whereby they induce the apoptotic cell death program and inhibit cell growth through IGF-dependent and -independent mechanisms (24–26).

Using co-immunoprecipitation (co-IP) and biolayer interferometry (BLI), the present study uncovered IGFBP-4, along with IGFBP-2 and -3, as binding partners for AIM. An apoptosis assay showed that AIM inhibited apoptosis through disrupting IGFBP-4 binding to IGF-I. These data provide the first evidence for AIM binding to IGFBPs, suggesting a potential mechanism for AIM-regulated cell survival.

## Materials and methods

### Materials

Rabbit anti-HA polyclonal antibody (0906-1) and mouse anti-His monoclonal antibody (M0812-3) were purchased from Hangzhou HuaAn Biotechnology Co. (Hangzhou, China). Horseradish peroxidase (HRP)-conjugated anti-mouse immunoglobulin G (IgG) (SA00001-1) and anti-rabbit IgG (SA00001-2) were purchased from the ProteinTech Group (Chicago, IL, USA). HisTrap HP columns and PD-10 desalting columns were purchased from Amersham Pharmacia Biotech, Inc. (Piscataway, NJ, USA). Anti-FLAG antibody (F3165), mouse anti-HA monoclonal antibody (H9658), control IgG (M5284) and Coomassie blue R-250 were purchased from Sigma (St. Louis, MO, USA).

### Cell culture

The 293 cells (ATCC, Manassas, VA, USA) were maintained in high-glucose Dulbecco's modified Eagle's medium (DMEM) supplemented with 10% (v/v) fetal bovine serum (FBS) (Invitrogen, Carlsbad, CA, USA). TAMH cells [a mouse hepatocyte cell line; provided by Christopher C. Franklin, Department of Pharmaceutical Sciences, University of Colorado Denver (UCD), Denver, CO, USA] were grown in serum free DMEM/Ham's F12 (Invitrogen) supplemented with 5 mg/ml insulin, 5 mg/ml transferrin, 5 ng/ml selenium (Collaborative Biomedical Products, Bedford, MA, USA), 100 nM dexamethasone, 10 mM nicotinamide and 0.1% (v/v) gentamicin (Invitrogen). *Drosophila* Schneider cells (S2 cells) were cultured in Schneider's *Drosophila* medium (Sigma) supplemented with 10% (v/v) FBS. Cultures were maintained in a humidified incubator with 5% carbon dioxide and 95% air at 37°C.

### Protein expression and purification

#### Eukaryotic expression system

The *AIM* gene was cloned into a modified pMT/BiP vector (Invitrogen), which contained 6 histidines and BirA enzyme substrate peptide (BSP, GGGLNDIFEAQKIEWHE) at the amino terminus. The recombinant plasmid and pCoHygro (19:1 ratio) were used to co-transfect S2 cells using the calcium phosphate method. After 4 weeks of culture in Schneider's *Drosophila* medium supplemented with 10% FBS and 300 *µ*g/ml hygromycin-B (Invitrogen), hygromycin-B resistant cells were selected. For large-scale production of soluble AIM protein, stably transfected S2 cells were cultured in EX-CELL 420 medium supplemented with 50 *µ*g/ml gentamycin (Sigma) at 28°C. Expression was induced with 0.5 mM CuSO_4_ for 7 days. Cells were removed by centrifugation at 1,000 × g, 4°C for 10 min. The supernatant was exchanged with buffer containing 50 mM NaH_2_PO_4_ and 500 mM NaCl (pH 8.0). The resultant solution was passed through a 0.22-*µ*m filter, supplemented with 5 mM imidazole, and subsequently purified by Ni-NTA chromatography. Proteins were stored at −80°C following determination of the concentration by the Bradford assay.

#### Prokaryotic expression system

The *AIM* gene carrying a HA tag sequence at the 5′-terminus was cloned into a pET28a vector (Novagen, Madison, WI, USA), containing 6 histidines at the amino terminus. The *IGFBP-4* gene was cloned into a pET28c vector (Novagen), containing 6 histidines at the amino terminus. The *Escherichia coli* (*E. coli*) strains, BL21 (DE3) transformed with the expression plasmid, including His-HA-tagged AIM or His-IGFBP-4, were induced with 1 mM isopropyl β-D-thiogalactopyranoside to express His-HA-AIM or His-IGFBP-4 protein. The proteins were purified using Ni-NTA affinity chromatography (Qiagen, Valencia, CA, USA) and stored at −80°C following determination of concentration by the Bradford assay.

#### Western blot assay

Samples were fractionated by sodium dodecyl sulfate-polyacrylamide gel electrophoresis (SDS-PAGE) and the separated proteins were electrophoretically transferred onto the nitrocellulose membranes (Bio-Rad, Hercules, CA, USA). Non-specific binding was blocked with 0.05% Tween-20 in phosphate-buffered saline (PBST) containing 5% non-fat milk for 1 h at room temperature. The membranes were subsequently incubated overnight at 4°C with antibodies against His tag or HA tag in PBST containing 1% non-fat milk at the dilutions specified by the manufacturers. Following 3 washes with PBST, the membranes were incubated with the HRP-conjugated secondary antibodies at 1:5,000 dilution in PBST containing 1% non-fat milk for 1 h at room temperature. The membranes were subsequently washed 3 times with PBST and the protein bands were detected with a western blotting detection system.

#### Co-IP

The *AIM* or *IGFBP* 1–6 genes were cloned into a cytomegalovirus promoter-based vector-pRK containing a 5′-HA or 5′-FLAG-tag. The plasmids were transiently transfected into the 293 cells (2×10^6^) using the calcium phosphate method. After 24 h, the transfected cells were lysed with 1 ml of lysis buffer [20 mM Tris-Cl (pH 7.5), 150 mM NaCl, 1% Triton X-100, 1 mM ethylenediaminetetraacetic acid, 1 mM phenylmethylsulfonyl fluoride, 2 mM Na_3_VO_4_, 20 mM NaF, 10 *µ*g/ml aprotinin and 10 *µ*g/ml leupeptin]. Cell lysate (0.4 ml) was incubated with the appropriate monoclonal antibody or control IgG, as well as 20 *µ*l of a 1:1 slurry of GammaBind Plus-Sepharose (GE Healthcare, Logan, UT, USA). After an overnight incubation at 4°C, the sepharose beads were washed 3 times with 1 ml lysis buffer. The precipitates were fractionated by SDS-PAGE, and western blot analysis was performed. All the immunoprecipitation experiments were repeated ≥3 times, and similar data were obtained.

#### BLI

The interaction between AIM and IGFBP-4 was measured using Bio-Layer Interferometry on Octet RED (ForteBio, Menlo Park, CA, USA). All the interaction analyses were conducted at 25°C in PBS buffer unless stated. IGFBP-4 was purified from *E. coli* as described above and subsequently labeled with biotin (Thermo Fisher Scientific, Waltham, MA, USA), which is optimal for binding and immobilizing target proteins on superstreptavidin (SA) biosensors (ForteBio) for studying protein-protein interactions. Biotinylated IGFBP-4 was separated and loaded onto SA biosensors for 300 sec to ensure saturation. The 96-well microplates used in the Octet were filled with 200 *µ*l of sample or buffer per well and agitated at 800 × g. The loaded biosensors were washed in buffer for 120 sec and transferred to the wells containing AIM at concentrations of 400, 200, 100, 50, 25 and 12.5 nM in buffer, respectively. The association was observed for 240 sec and dissociation was observed for 300 sec for each protein of interest in the sample diluent. A parallel set of superstreptavidin biosensors was prepared with biotinylated streptavidin to act as a control. Kinetic parameters (*K*_on_ and *K*_off_) and affinities (*K*_D_) were calculated from a non-linear global fit of the data between IGFBP-4 and AIM using the Octet software. Independent measurements were performed ≥3 times.

#### Caspase-3/7 activity

The TAMH cells were plated in 96-well plates at a density of 1×10^4^ cells/well for 24 h. Cells were subsequently washed with DMEM/F12 medium and serum-starved overnight. Certain cultures were treated with IGF-I (20 ng/ml) in the presence or absence of IGFBP-4 (500 ng/ml) and AIM (2 *µ*g/ml) for 6 h before the supernatant and cells were harvested. Cells were lysed for 20 min on ice, and subsequently incubated with caspase-3/7 substrate (Ac-DEVE-AMC, 20 *µ*M) for 1 h at 37°C. Fluorescence intensity was measured using excitation wavelength of 380 nm and an emission wavelength of 460 nm.

#### Statistical analysis

All the conditions were performed in triplicate, and the reported values are representative of 3 independent experiments. All the values are expressed as the mean ± standard deviation of 3 parallel measurements. Data were analyzed by Student's t-test and P<0.05 was considered to indicate a statistically significant difference.

## Results

### Identification of binding partners of AIM

**Figure 1:**
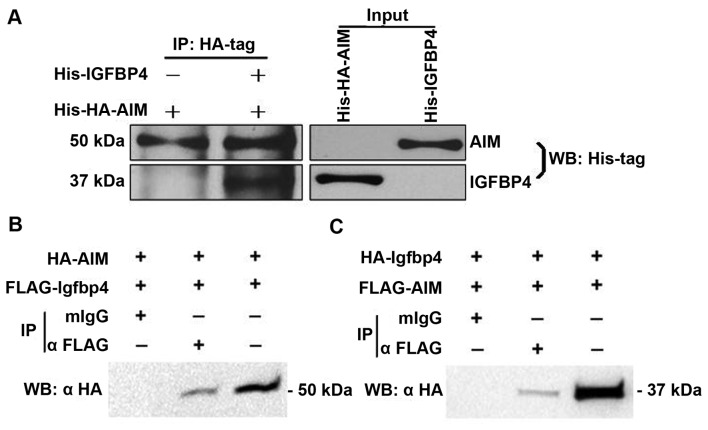
Apoptosis inhibitor of macrophages (AIM) can interact with insulin-like growth factor binding protein-4 (IGFBP-4). (A) AIM can interact with IGFBP-4 *in vitro*. The purified His-HA-AIM was pre-incubated with His-IGFBP-4 for 2 h, and was subjected to co-IP using the anti-HA tag polyclonal antibody. The immunoprecipitates were analyzed by sodium dodecyl sulfate-polyacrylamide gel electrophoresis and western blot analysis using anti-His tag antibody. (B and C) AIM can interact with IGFBP-4 in the 293 cells. The 293 cells were co-transfected with either HA-AIM and FLAG-IGFBP-4 or HA-IGFBP-4 and FLAG-AIM. The co-IP experiments were conducted using anti-FLAG antibody. Western blot analysis was performed using the HA monoclonal antibody. The rightmost lane in each panel represents the total un-precipitated sample control. IP, immunoprecipitation; WB, western blot.

Our yeast two hybrid screening for binding partners of AIM identified IGFBP-4 as a potential candidate. To further confirm that AIM binds to IGFBP-4, the proteins His-HA-AIM and His-IGFBP-4 were purified from transformed *E. coli* cells, respectively, and subsequently, the binding activity of AIM to IGFBP-4 was assessed using the *in vitro* co-IP assay. The results showed that His-IGFBP-4 appeared in immunoprecipitates of His-HA-AIM bound to beads with anti-HA tag antibody (Fig. 1A), demonstrating that the recombinant AIM and IGFBP-4 purified from *E. coli* could interact with each other *in vitro*.

In order to identify the interaction between AIM and IGFBP-4 in mammalian cells, 293 cells were co-transfected with expression vectors encoding HA-AIM, FLAG-AIM, HA-IGFBP-4 and FLAG-IGFBP-4. The interaction between AIM and IGFBP-4 was determined by the co-IP assay. As shown in Fig. 1, HA-AIM was clearly detected in the immunoprecipitates of Flag-IGFBP-4 bound to beads with anti-Flag antibody (Fig. 1B). Reciprocally, HA-IGFBP-4 was also readily precipitated with Flag-AIM on beads with anti-Flag antibody (Fig. 1C).

**Figure 2:**
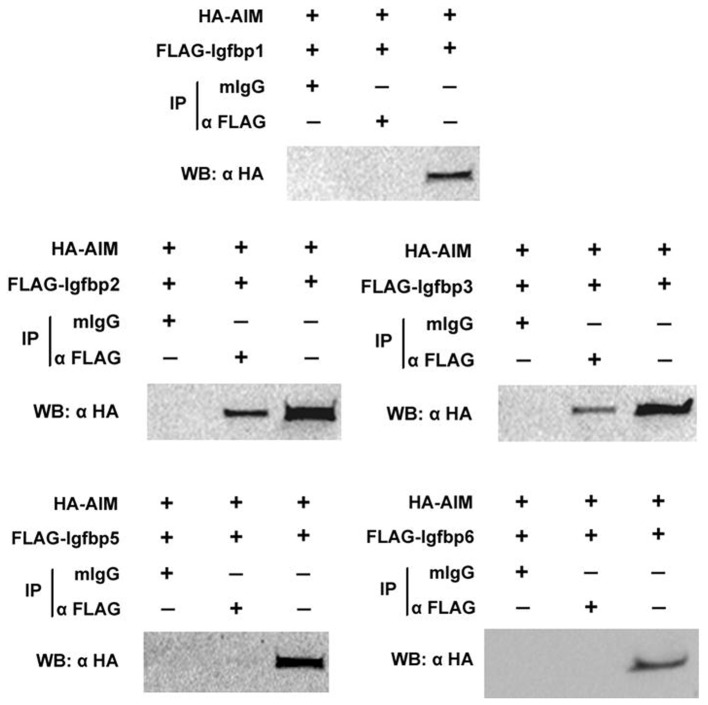
Apoptosis inhibitor of macrophages (AIM) interacts with insulin-like growth factor binding protein-2 (IGFBP-2) and IGFBP-3, but not IGFBP-1, -5 or -6. The 293 cells were co-transfected with HA-AIM and FLAG-IGFBPs (1, 2, 3, 5 or 6) for 24 h and subsequently lysed. The cell lysis was subjected to the co-immunoprecipitation and western blot (WB) assays. The rightmost lane in each panel represents the total un-precipitated sample control.

The family of IGFBPs consists of ≥6 isoforms that have been well characterized (17). Subsequently, whether AIM could interact with other IGFBP family members was investigated. The 293 cells were co-transfected with HA-AIM and Flag-IGFBPs (1, 2, 3, 5 or 6) for 24 h. Cell extracts from the 293 cells were immunoprecipitated with anti-Flag antibody or control IgG. Western blot analysis revealed that AIM can interact with IGFBP-2 and IGFBP-3, but not IGFBP-1, IGFBP-5 or IGFBP-6 (Fig. 2).

### Evaluation of binding activity between AIM and IGFBP-4

**Figure 3:**
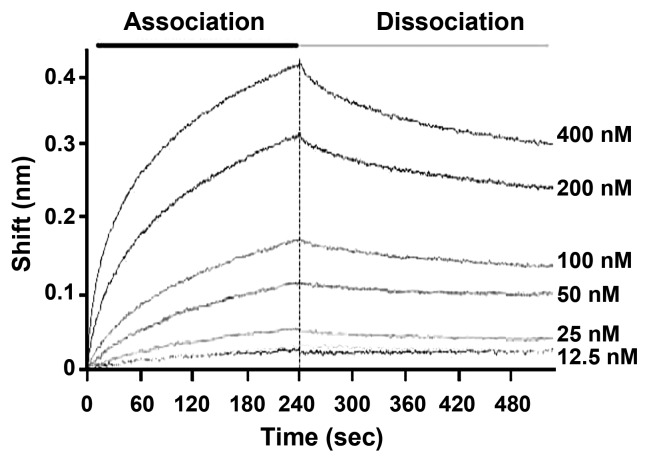
Kinetic analysis of the interaction between apoptosis inhibitor of macrophages (AIM) and insulin-like growth factor binding protein-4 (IGFBP-4) by biolayer interferometry. The Super Streptavidin biosensor tips of the ForteBio Octet RED 96 were coated with biotinylated IGFBP-4. The biosensor tips were dipped in increasing concentrations of AIM (indicated on the right side of each binding curve) to measure binding of AIM to IGFBP-4 (left side of the graph) and subsequently moved to wells containing buffer to measure dissociation rates (right side of the graph). A representative experiment of three replicates is shown.

To obtain the kinetic parameters of the interaction between AIM and IGFBP-4, BLI was employed to assess the rates of association and dissociation. IGFBP-4 was biotin-labeled and captured on the SA sensor chips, and kinetic parameters were determined for AIM as analytes (Fig. 3). The results show that AIM rapidly associates with biotinylated IGFBP-4 bound to SA-biosensors through the fast association rate constant (*K*_on_) of 3.96±0.04×10^4^ Ms^−1^. By contrast, the dissociation of AIM from biotinylated IGFBP-4 on SA biosensors appeared to be slow, evident from the dissociation rate constant (*K*_off_) of 9.99±0.11×10^−4^ s^−1^. The equilibrium dissociation constant (*K*_D_) of AIM was 2.53±0.06×10^−8^ M, which was calculated from the ratio of the rate constant, *K*_off_/*K*_on_ (Table I). These data reveal a strong association between AIM and IGFBP-4.

### AIM inhibits IGFBP-4-mediated apoptosis

**Figure 4:**
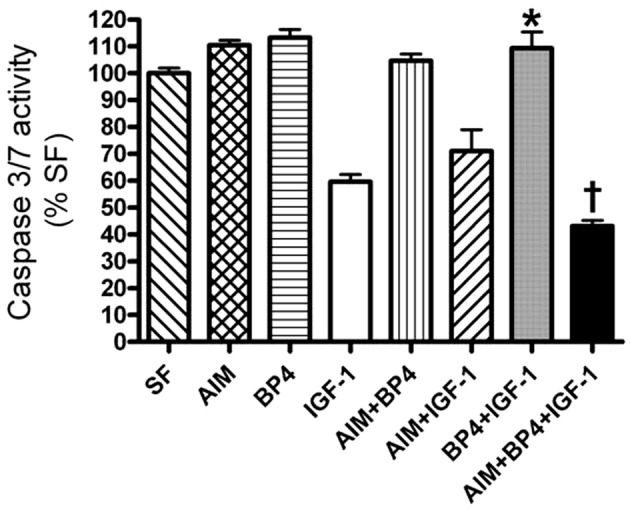
Apoptosis inhibitor of macrophages (AIM) inhibits apoptosis through disrupting insulin-like growth factor binding protein-4 (IGFBP-4) binding to IGF-I. TAMH cells (1×10^4^/well) were plated in 96-well plates for 24 h. Cells were subsequently washed with Dulbecco's modified Eagle's medium/F12 medium and serum-starved overnight. IGF-I (20 ng/ml), IGFBP-4 (500 ng/ml) or AIM protein (2 *µ*g/ml) were incubated in the culture for 6 h before supernatants and cells were harvested. Cells were subsequently incubated with caspase-3/7 substrate and fluorescence intensity was measured. ^*^P<0.05 compared with IGF-I alone; ^†^P<0.05 compared with the group of BP4 + IGF-I. BP4, IGFBP-4; SF, serum free.

Previous studies have demonstrated that IGFBPs modulate the biological activity of IGF-I to inhibit cell growth and proliferation (24,27–29). Thus, we hypothesized that AIM inhibited apoptosis by binding to IGFBP-4, thus increasing the binding of IGF-I to IGF receptors. To examine this hypothesis, TAMH cells were treated with IGF-I in the presence and absence of IGFBP-4 and AIM, and apoptosis was examined by measuring the caspase-3/7 activities. The caspase-3/7 activity induced by serum starvation in TAMH cells was significantly decreased when the cells were treated with IGF-I, indicating an anti-apoptotic and pro-survival effect of IGF-I (Fig. 4). The effect was abrogated by IGFBP-4, as the caspase-3/7 activity returned to the levels of serum-starved cells. This result is consistent with a previous report that IGFBP-4 inhibited the pro-survival activity of IGF-I (29). Compared with the caspase-3/7 activity in cells treated with IGFBP-4 + IGF-I, the caspase activity in cells treated with the combination of AIM, IGFBP-4 and IGF-I was markedly reduced. These results revealed that the mechanism of AIM inhibiting apoptosis was partly through disrupting IGFBP-4 binding to IGF-I.

## Discussion

AIM, produced and secreted by tissue macrophages, has been indicated in a broad spectrum of biological functions, attributable to its anti-apoptotic effects on macrophages and other cell types (3,5). However, the molecular mechanism by which AIM regulates apoptosis is not clear. The present data demonstrate that AIM binds to IGFBP-4, which may explain the underlying mechanism of AIM-mediated anti-apoptotic function.

AIM acts as an immune regulator by inhibiting immune cell apoptosis. AIM inhibits apoptosis of monocytes, T cells, NKT cells and CD4/CD8 double-positive thymocytes undergoing maturation in the thymus, and the loss of AIM promotes cell apoptosis at the inflammatory sites (3,5). Recent investigations from animal models have shown that AIM appears to be multifunctional and is effective in cell types other than immune cells, including adipocytes and epithelial cells (13–15,30). Increasing evidence has shown that AIM has key roles in the pathogenesis of multiple diseases, including atherosclerosis, metabolic diseases, inflammation, infection and cancer by supporting the survival of macrophages (6,8,10–13,31). Thus, AIM may be a therapeutic target in these diseases (30).

With regard to the anti-apoptosis function of AIM, it has been suggested that Stat3, Erk1/2 and p38 signaling pathways may be involved (14). However, the underlying molecular mechanism accounting for the action of AIM is not understood, as the binding partners of AIM are unknown. Previous data have shown that the circulating level of AIM is dependent on its association with the IgM pentamer to increase the stabilization in blood (32). The present data demonstrate that AIM binds to IGFBP-4 and that this interaction may have an important role in mediating the anti-apoptotic function of AIM. All IGFBPs share a highly conserved structure that is generally described as consisting of three distinct domains of approximate lengths as follows: Highly conserved cysteine-rich N and C domains and a central linker domain, unique to each IGFBP species. IGFBPs exist in the circulation in the free form or in complexes with IGFs, thereby prolonging their half-lives and modulating their biological functions in target cells (17,23). The present results revealed that AIM can interact with IGFBP-2, -3 and -4, but not IGFBP-1, -5 or -6 (Fig. 2). According to the evolution assay of the IGFBPs family, they can be divided into 2 main subgroups. One cluster includes IGFBP-1, -2 and -4 as they are more closely associated with each other, the other includes IGFBP-3, -5 and -6 (33). Furthermore, there are significant similarities between the IGFBP-2-NMR crystal structure and IGFBP-4-X-ray crystal structure (23,34,35). The majority of the residues involved in binding to IGF-I were conserved between N-BP-2 and N-BP-4; these two residues show that the N- and C-termini are in close contact. Due to the structural similarities, AIM may interact with IGFBP-2 and -4 in a similar manner. Although IGFBP-1 belongs to the same group as IGFBP-2 and -4, it is not able to bind to AIM. By contrast, IGFBP-3 can interact with AIM despite belonging to a different group. Further detailed investigations are warranted to identify the critical domains in the IGFBP proteins that are critical in binding to AIM.

Among all the IGFBPs, IGFBP-4 has been predominately associated with counteracting the pro-survival and pro-proliferative effects of IGF. IGFBP-4 decreases cell proliferation and DNA synthesis, as well as induces apoptosis in a cell type- and tissue-specific manner (36,37). Sitar *et al* (23) reported the high-resolution X-ray structure of a complex of the N- and C-terminal domains of IGFBP-4 bound to IGF-I, which provided the structural basis for the inhibition of IGFs by IGFBP-4. The N-terminal domain of IGFBP-4 contains pivotal IGF-binding residues, rendering high-affinity binding to IGF and partially masking the IGF residues responsible for the type 1 IGF receptor binding. The C-terminal domain also contributes to blocking of the IGF-I receptor-binding region of IGF-I. The central domain of the IGFBP-4 contains proteolytic cleavage sites. On these sites, the IGFBP-4 protease specifically cleaves IGFBP-4 into fragments with low affinity for IGF-I, leading to IGF release (17). The present data demonstrate that AIM can directly bind to IGFBP-4 and reverse the pro-apoptotic effect of IGFBP-4. We hypothesize that the binding of AIM to IGFBP-4 may reduce the affinity of its N- and/or C-terminal domains that bind with IGF. The competition for IGFBP-4 binding by AIM releases IGF, thereby promoting IGF binding to IGF receptors and thus IGF signaling.

In conclusion, to the best of our knowledge, this study provides the first evidence that AIM binds to IGFBP-2, -3 and -4. The data suggest that this interaction between AIM and IGFBP-4 may contribute to the mechanism of AIM-mediated anti-apoptosis effect. These findings may provide valuable information regarding the mechanism of apoptosis regulation by AIM.

## Figures and Tables

**Table I:** Affinity and rate constants for the interaction between AIM and IGFBP-4.

Interaction proteins	*K*_D_ (×10^−8^ M)	Rate constants		R^2^
		*K*_on_ (×10^4^ Ms^−1^)	*K*_off_ (×10^−4^ s^−1^)	
AIM IGFBP-4	2.53±0.06	3.96±0.04	9.99±0.11	0.988695

Fast association rate constant (*K*_on_) and the dissociation rate constant (*K*_off_) were averaged over three independent experiments. Data are presented as means ± standard deviation of three biological replicates. R^2^ is the coefficient of determination estimating the goodness of curve fit reported by ForteBio Data Analysis software version 8.0. All the measurements were performed at 25°C. AIM, apoptosis inhibitor of macrophages; IGFBP-4, insulin-like growth factor binding protein-4.

## Abbreviations

AIM: apoptosis inhibitor of macrophage
LXR: liver X receptor
IGF: insulin-like growth factor
IGFBP: insulin-like growth factor binding protein
